# Hemopoietic-specific *Sf3b1*-K700E knock-in mice display the splicing defect seen in human MDS but develop anemia without ring sideroblasts

**DOI:** 10.1038/leu.2016.251

**Published:** 2016-10-21

**Authors:** A Mupo, M Seiler, V Sathiaseelan, A Pance, Y Yang, A A Agrawal, F Iorio, R Bautista, S Pacharne, K Tzelepis, N Manes, P Wright, E Papaemmanuil, D G Kent, P C Campbell, S Buonamici, N Bolli, G S Vassiliou

**Affiliations:** 1 Haematological Cancer Genetics, Wellcome Sanger Institute, Hinxton, Cambridge, UK; 2 H3 Biomedicine, Inc., Cambridge, MA, USA; 3 Cambridge Stem Cell Institute, Cambridge, UK; 4 Malaria Programme, Wellcome Sanger Institute, Hinxton, Cambridge, UK; 5 European Bioinformatics, Institute, Hinxton, Cambridge, UK; 6 LIMS Compute and Infrastructure, Wellcome Sanger Institute, Hinxton, Cambridge, UK; 7 Department of Pathology, Cambridge University Hospitals NHS Trust, Cambridge, UK; 8 Memorial Sloan Kettering Cancer Center, New York, NY, USA; 9 Cancer Genome Project, Wellcome Trust Sanger Institute, Hinxton, Cambridge, UK; 10 Dipartimento di Oncologia ed Onco-Ematologia, Universita' degli Studi di Milano, Milano, Italy; 11 Dipartimento di Ematologia ed Onco-Ematologia Pediatrica, Fondazione IRCCS Istituto Nazionale dei Tumori, Milano, Italy; 12 Department of Haematology, University of Cambridge, Cambridge, UK; 13 Department of Haematology, Cambridge University Hospitals NHS Foundation Trust, Cambridge, UK

## Abstract

Heterozygous somatic mutations affecting the spliceosome gene *SF3B1* drive age-related clonal hematopoiesis, myelodysplastic syndromes (MDS) and other neoplasms. To study their role in such disorders, we generated knock-in mice with hematopoietic-specific expression of *Sf3b1-K700E*, the commonest type of *SF3B1* mutation in MDS. *Sf3b1*^*K700E/+*^ animals had impaired erythropoiesis and progressive anemia without ringed sideroblasts, as well as reduced hematopoietic stem cell numbers and host-repopulating fitness. To understand the molecular basis of these observations, we analyzed global RNA splicing in *Sf3b1*^*K700E/+*^ hematopoietic cells. Aberrant splicing was associated with the usage of cryptic 3′ splice and branchpoint sites, as described for human *SF3B1* mutants. However, we found a little overlap between aberrantly spliced mRNAs in mouse versus human, suggesting that anemia may be a consequence of globally disrupted splicing. Furthermore, the murine orthologues of genes associated with ring sideroblasts in human MDS, including *Abcb7* and *Tmem14c*, were not aberrantly spliced in *Sf3b1*^*K700E/+*^ mice. Our findings demonstrate that, despite significant differences in affected transcripts, there is overlap in the phenotypes associated with *SF3B1-K700E* between human and mouse. Future studies should focus on understanding the basis of these similarities and differences as a means of deciphering the consequences of spliceosome gene mutations in MDS.

## Introduction

The myelodysplastic syndromes (MDS) are clonal blood disorders characterized by dysplastic hematopoiesis and blood cytopenias.^1^ Somatic mutations affecting the genes *SF3B1*, *SRSF2, U2AF1*, *ZRSR2* and *LUC7L2,* which code for components of the spliceosome, are found in more than half of MDS patients.^2, 3^ *SF3B1* mutations are the commonest among these and are associated with the presence of bone marrow ringed sideroblasts^2^ and a favorable prognosis.^4, 5^ The mutations are missense, heterozygous and cluster strongly within HEAT domains 4–8 of the protein, suggesting that they may be neomorphic gain-of-function variants.^2^ Studies analyzing the subclonal composition of MDS indicate that *SF3B1* mutations represent early/initiating lesions in MDS evolution,^5, 6, 7^ and this was corroborated by their recent identification in hematopoietic cell clones found in at least 2% of otherwise healthy individuals aged 70 years or older.^8^ Furthermore, in a significant proportion of *SF3B1*-mutant MDS this is the only identifiable driver mutation,^7, 9^ suggesting that in the right context these mutations can impart a substantial fitness advantage on hematopoietic stem cells. However, the molecular effects of *SF3B1* mutations and the mechanisms through which they drive clonal expansion and dyserythropoiesis remain obscure.

RNA splicing takes place at splice sites (ss) located at the 5′ and 3′ ends of introns, after the recognition of consensus nucleotide sequences by the spliceosome machinery. The U1 small nuclear ribonucleoprotein particle (snRNP) recognizes the 5′ ss, and at the 3′ ss the polypyrimidine (Py) tract and an invariant AG dinucleotide located at the intron-exon junction are recognized by the U2AF1–U2AF2 complex, while the proximal branch point sequence (BPS) is identified by the U2 snRNP, of which SF3B1 is a component.^10^ MDS cases associated with *SF3B1* mutations show a distinct gene expression profile, including dysregulation of genes required for heme biosynthesis, such as *ALAS2* and *ABCB7*.^11, 12^ Furthermore, it was recently demonstrated in primary human cancers and cell lines that *SF3B1* mutations are associated with aberrant splicing through recognition of alternative 3′ ss located around 10–25 nt upstream of the canonical 3′ ss.^13^ However, the consequences of these aberrations and their impact on hematopoiesis are not well understood.

Here, to investigate the molecular and phenotypic consequences of *SF3B1* mutations on hematopoiesis, we generate and study an accurate conditional knock-in mouse model of *SF3B1*-K700E, the commonest mutation in MDS. Our findings show that while the sequence features of the aberrant splicing are extremely similar between mouse and human,^13^ the genes affected differ significantly. Despite these marked differences, *Sf3b1*^K700E/+^ mice did go on to develop progressive anemia, a central feature of *SF3B1*-mutant MDS. However, other characteristic features of *SF3B1*-mutant MDS such as ring sideroblasts and a stem cell growth advantage were not observed. Our results propose that the anemia associated with mutant *SF3B1* may be a consequence of globally disrupted splicing rather than effects on particular genes while other features of *SF3B1*-mutant MDS may be gene-specific.

## Materials and methods

### Generation of the *Sf3b1*-K700E targeting construct

The targeting construct was generated using gateway and recombineering recombination technologies as described previously.^14^ Briefly, a pool of two BACs containing the region of interest of the *Sf3b1* locus, RP24-64H9 and RP24-439B17, were used to generate the gateway-adapted intermediate plasmid by inserting the ‘U' and ‘G' cassettes.^14^ The latter were generated by PCR and the ‘U' cassette inserted into the BAC first so that the ‘G' cassette then retrieved the relevant portion of the *Sf3b1* locus into a gateway-adapted intermediate plasmid.^15^ The endogenous exon 15 was then replaced with a synthetic one bearing the A>G mutation encoding for the K700E variant. The synthetic DNA was made by GeneArt (Life Technologies, Regensburg, Germany), and was subcloned into the intermediate vector exploiting two *Kpn*I sites in introns 14 and 16 (Supplementary Figure 1). The splice trap cassette encoding an Engrail-2 splice acceptor site, codon-optimized exons 12–15, intron 15, codon-optimized exons 15–19, intron 19 and native exons 20–25 (including the 3′ UTR) was synthesized by GeneArt (Life Technologies) and subcloned into the pL1L2_BactP vector previously modified to remove the EnSA-IRES-lacZ-pA-LoxP and subsequently the 3′ *LoxP* site by digestion. The final targeting vector was built using the two vectors above and the pL3L4-DTA vector^14^ in a multi-vector gateway reaction, and was linearized prior to electroporation using the *Asis*I restriction site (Supplementary Figure 2). Sequences of primers used to generate and validate the targeting construct are given in Supplementary Table 1.

### ES cell culture and validation of clones

JM8 ES cells were used for electroporation of the final targeting vector. Cells were grown for 9 days after electroporation in a feeder-coated plate with DMEM medium (Invitrogen, Hempstead, UK) supplemented with 10% FBS (Biosera, UK), 1% BME (Sigma-Aldrich, Haverhill, UK), 1000 U/ml leukemia inhibitory factor (ESGRO, Millipore, UK) and G418 (Sigma-Aldrich) 180 μg/ml. Clones were picked and replated in a 96-well plate with feeders, split in replica plates after 5 days, and lysis for DNA extraction was performed after seven additional days. Clones were screened by 5′ and 3′ long-range PCR (Supplementary Figure 3). Three clones with successful 5′ and 3′ amplification, were and all showed that the splice-trap cassette was indeed functional (Supplementary Figure 4a and b). ES cell clones post exposure to FLP and Cre recombinases were validated for excision of the positive selection marker and cDNA splice-trap cassette, respectively (Supplementary Figure 4c). To confirm that the K700E mutation was not expressed in basal conditions but only after Cre expression, cDNA from clone D9 pre- and post- excision of the splice trap cassette was sequenced (Supplementary Figure 4d).

### Hematological measurements and mouse survival

Blood counts were performed using a VetABC analyzer (Horiba ABX, Montpellier, France). Mice were monitored daily and culled if they showed signs of illness or suffering. All animal studies were performed according to the Animals Scientific Procedures Act 1986 (ASPA), as recently revised to transpose European Directive 2010/63/EU on the protection of animals used for scientific purposes. Mouse cohort numbers were estimated to detect (*P<*0.05) a difference in survival of 2 months or greater with a power of 0.8, assuming a standard deviation of 2.2 months (http://www.biomath.info/power/ttest.htm).

### Competitive hematopoietic progenitor transplantation

C57BL/6 mice (*n*=4 per group) were irradiated with a lethal dose of 2 × 500 rad. They were then injected into the tail vein with a combination of 250 000 lineage negative bone marrow cells (lin^−^) from C57BL/6.SJL (*Sf3b1*^+/+^, CD45.1) mice and 250 000 lin^−^ cells from *Sf3b1*^*K700E/+*^ (CD45.2) mice plus 250 000 whole bone marrow (BM) rescue cells from C57BL/6/6.SJL (CD45.1/CD45.2) mice. Control animals were injected with 1  ×  10^6^ whole BM cells from C57BL/6/6.SJL (CD45.1/CD45.2). Blood counts and FACS analysis were performed after 1, 2 and 4 months. Samples were stained with CD45.1-APC (Becton Dickinson, Oxford, UK) and CD45.2-FITC (Becton Dickinson) antibodies run on Becton Dickinson LSFR Fortessa and analyzed with Flowjo 7.6.5 (FlowJo, LLC, San Diego, CA, USA).

### Immunophenotyping

For mouse phenotyping peripheral blood and bone marrow from femurs were collected from animals of different genotypes and processed as previously described.^16^ For flow analysis, samples were stained with Gr1-PE (Miltenyi Biotec, Woking, UK), CD11b (Mac1)-FITC (Becton Dickinson), CD71-PE (Becton Dickinson), Ter119-Fitc (Becton Dickinson). For analysis of progenitor populations BM cells were lysed with Red Blood Cell buffer (NH4, Cl) and then enriched with the EasySep Hematopoietic Progenitor Cell Isolation Kit (StemCell Technologies, Cambridge, UK) followed by staining with following antibodies Sca1-PB, c-KIT-Acy7, CD34-FITC, Flk2-PeCy5, Cd16/32-PE, CD150-PeCy7, CD48-APC.

### RNA isolation and analysis

Whole BM cells were harvested from femurs of *Sf3b1^+/+^* and *Sf3b1^K700E/+^* animals. lin^−^ cells were isolated with MACS separation system (Miltenyi Biotec) following standard protocol. RNA from whole BM was isolated using an RNA isolation Kit (Qiagen, Manchester, UK) and RNA from lin^−^ cells with PicoPure RNA Isolation System (Thermo Fisher, Hempstead, UK). Sequencing was performed on the Hiseq2000 V4 sequencing platform (llumina, San Diego, CA, USA). Sequence data have been deposited in ArrayExpress with the accession number E-ERAD-379. All RNA-seq data were analyzed as described previously.^13^ RNA-seq datasets have been deposited at the NCBI GEO and are available under accession number GEO:GSE72790. STAR alignment software was used to align raw sequence fragments to the hg19 human reference genome.^17^ Junction percent spliced-in (PSI) was calculated for any two or more splice junctions that share a junction boundary and have alternative end points by dividing the raw count of each junction species by the total sum of junction counts that also share that boundary. Intron retention was factored into PSI calculations by counting reads that aligned completely to exon–intron boundaries, defined as 6 basepair (bp) windows, 3 bp in the exon and 3 bp in the intron. We used a moderated *t*-test from the stastical package limma^18^ to determine the significance of PSI measurements. We used the Gencode v19 transcriptome annotation to evaluate whether junction boundaries were equivalent to known exon boundaries (that is, junction novelty). Gene counts were obtained using the Sailfish package,^19^ and differential gene expression calculated using edgeR.^20^ All *P*-values were corrected using the Benjamini–Hochberg procedure and *q*-values <0.05 were considered significant.

### Nested PCR for lariat sequencing

Gene-specific reverse transcription of the endogenous lariat region was done using Superscript IV (Invitrogen) and primer C (Supplementary Table 2). The resulting cDNA was used for nested PCR reactions using Platinum Taq DNA polymerase (Invitrogen) and two sets of primers, outer primers (C and D) followed by inner primers (A and B) (Supplementary File 3). The PCR product from the second reaction was run on 2.0% agarose gel and gel-extracted for Sanger sequencing. The sequencing data were analyzed using Mutation Surveyor software.

### Statistical analyses

Blood counts and cellular compartment sizes were compared using unpaired *t*-test. Mouse survival was compared using the Kaplan Meier estimator. Adenine counts near canonical vs aberrant splice junctions and expression levels of transcripts that were NMD-predicted vs non-NMD predicted were compared using the Kruskal *H* test (Figure 3). Comparison of RNA-seq transcript levels was performed using edgeR^20^ (Figure 4).

## Results

**Figure 1:**
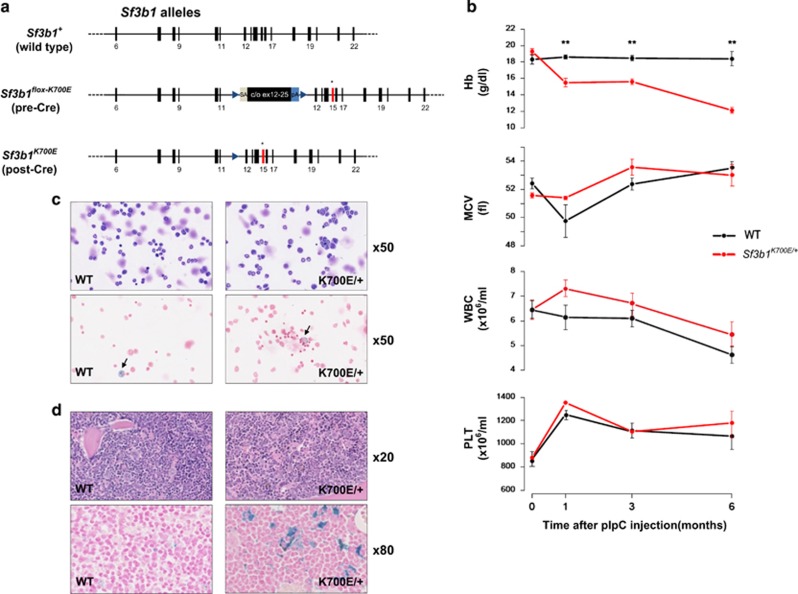
*Sf3b1*^*K700E/+*^ mice develop progressive normocytic anemia without ring sideroblasts. (**a**) *Sf3b1* alleles in study mice. The conditional (pre-Cre) allele contains a floxed splice trap cassette inserted within intron 11 of the gene. This begins with a splice acceptor site (SA) splicing in-frame into exons 12–25 of a partially codon-optimized (c/o) *Sf3b1* cDNA and ending with a polyadenylation signal (pA). This allele therefore expresses a chimeric mRNA, which is translated into the wild-type (WT) SF3B1 protein. Downstream of this, the endogenous exon 15 (red) was mutagenized to encode the K700E mutation. After Cre-mediated excision of the trap cassette, the recombined allele (post-Cre) expresses *Sf3b1-K700E*. (**b**) Serial blood counts highlight a progressive normocytic anemia in *Sf3b1*^*K700E/+*^ compared to age-matched WT mice. Platelet (PLT) and leukocyte counts (WBC) were not different between mutant and WT mice. (**c**) Examination of *Sf3b1*^*K700E/+*^ bone marrow cytospins showed no significant evidence of dysplasia (upper panel) and Perl staining demonstrated macrophage iron (arrows), but no ring sideroblasts (lower panel). (**d**) In keeping with the cytological findings, histological examination did not show significant dysplasia (upper panel), although Perl staining did reveal increased iron deposits in mutant bone marrow macrophages (lower panel). Hb, hemoglobin concentration; MCV, mean corpuscular volume.

Conditional *Sf3b1^flox-K700E/+^* mice were generated by targeted modification of the *Sf3b1* locus in JM8 mouse embryonic stem cells (ESCs)^14, 21^ (Figure 1a and Supplementary Figures 1 and 2). To ensure that normal mRNA splicing was retained, the targeted allele was designed to express normal SF3B1 protein via a part-codon optimized *Sf3b1* exon 12-25 cDNA (Figure 1a and Supplementary Figure 3). After germline transmission, a downstream neomycin cassette was removed *in vivo* through mating with Rosa26-FLPe mice,^22^ to generate the conditional *Sf3b1*^*flox−K700E*^ allele (Supplementary Figure 3). The *Sf3b1*^*K700E*^ mutant allele could then be expressed after Cre-*loxP* recombination (Figure 1a and Supplementary Figure 4).

To activate *Sf3b1*^*K700E*^ in hematopoietic stem cells, we crossed *Sf3b1^flox-K700E/+^* and *Mx1-Cre*^23^ mice, treated 20 double transgenic and 20 control (wild type and *Sf3b1^flox-K700E/+^*; *Mx1-Cre-*, henceforth referred as WT) 4- to 6-week-old mice with polyinosinic-polycytidylic acid (pIpC) and monitored the animals longitudinally. Monthly blood counts from mutant mice (henceforth referred to as *Sf3b1*^*K700E/+*^) showed a progressive normocytic anemia compared to wild type, but no significant differences in white blood cell (WBC) or platelet (PLT) counts (Figure 1b). Cytological examination of the bone marrow of *Sf3b1*^*K700E*/+^ mice showed no significant dysplasia and no ring sideroblasts (Figure 1c). Bone marrow (BM) histology did not reveal any significant morphological differences between WT and *Sf3b1*^*K700E/+*^ mice, but did show a moderate overall increase in iron deposits in the latter (Figure 1d). At a median follow-up of 83 weeks, mutant animals did not show altered survival or increased signs of illness when compared to WT controls (Supplementary Figure 5a).

**Figure 2:**
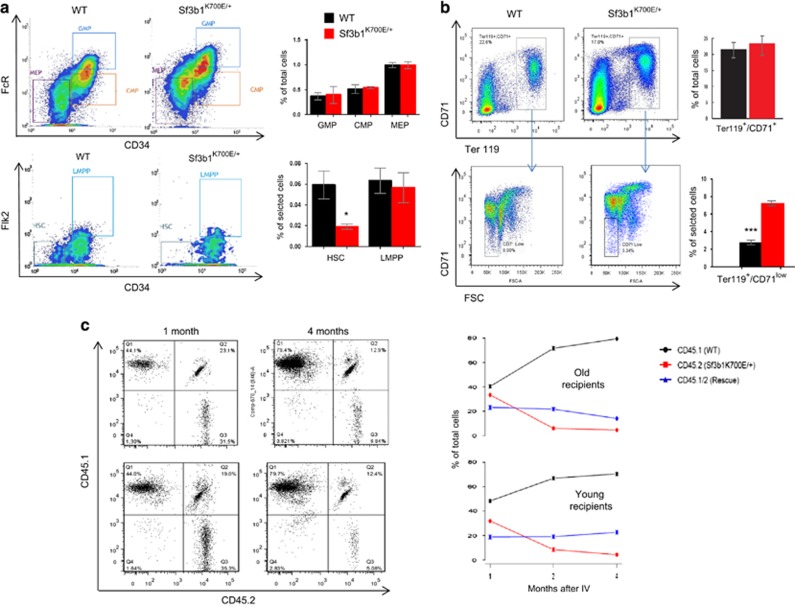
Effects of mutant *Sf3b1*^*K700E/+*^ on hemopoietic stem and progenitor cells. (**a**) Flow cytometric analysis of bone marrow cells from *Sf3b1*^*K700E/+*^ mice showed a significant decrease of phenotypically defined HSCs compared to WT animals, but no differences in LMPP, GMP, CMP or MEP progenitor numbers (*n* *=* 4). (**b**) Early erythroid cells (Ter119^+^/CD71^+^) were unchanged in number, but there was a reduction in mature Ter119^low^/CD71^+^ erythoid cells in *Sf3b1*^*K700E/+*^ animals. (**c**) Competitive transplantation of bone marrow lin^*−*^ cells from *WT* (CD45.1) and *Sf3b1*^*K700E/+*^ (CD45.2) into young (2 months) or old (1 year) syngeneic (C57BL/6) recipients (*n*=4 per group). Results shows that, after good initial engraftment (1 month), *Sf3b1*^*K700E/+*^ (CD45.2) progenitor cells were steadily outcompeted by co-transplanted *WT* (CD45.1) cells. Bars in (**a**, **b**) and datapoints in (**c**) show mean±standard error of the mean.

To investigate the consequences of *Sf3b1*^*K700E*^ expression on hematopoiesis in greater detail, we analyzed equal numbers of WT and mutant bone marrow cells harvested 4 weeks after pIpC injection by flow cytometry. We found that *Sf3b1*^*K700E/+*^ mice showed a significant decrease in the number of phenotypically defined hematopoietic stem cells (HSCs), but no change in the size of the LMPP, GMP, CMP and MEP progenitor compartments compared to WT littermates (Figure 2a). By contrast, *Sf3b1*^*K700E/+*^ animals showed a small but significant increase in the number of Gr1+/Mac1+ double-positive bone marrow cells (Supplementary Figure 5b), and in the erythroid compartment there were no differences in the frequency of Ter119+/CD71+ early erythroid cells, but a relative decrease of the more mature Ter119+/CD71^low^/FSC^low^ population^24^ (Figure 2b), indicative of impaired terminal erythroid differentiation similar to that observed in human MDS.^25^

Next, as *SF3B1-K700E* behaves like an initiating mutation in MDS^7^ and can also drive clonal hematopoiesis in elderly individuals,^8^ we wanted to investigate if it confers a fitness advantage to murine HSCs. To test this, we performed competitive transplantation assays in lethally irradiated syngeneic (C57BL/6) recipient mice. These experiments were performed in both young (2 months) and old (12 months) animals to investigate the possibility that age-related changes in recipient mice may influence the repopulating fitness of *Sf3b1^K700E/+^* mutant stem cells.^26^ Experimental mice were transplanted with an equal number (2.5  ×  10^5^) of bone marrow lineage negative (lin^*−*^) progenitor cells from WT (CD45.1) and *Sf3b1*^*K700E/+*^ (CD45.2) mice, as well as 2.5  ×  10^5^ whole bone marrow (rescue) cells from *Sf3b1*^*+/+*^ (CD45.1/CD45.2) animals. The control group was transplanted with rescue cells from whole bone marrow (CD45.1/CD45.2 = 1 × 10^6^). We found that although *Sf3b1*^*K700E/+*^ cells did show good engraftment at 1 month post transplantation, they displayed a significant decline in their white blood cell progeny after 4 months compared to WT cells. This was true for both young and old recipients (Figure 2c). Similar results were obtained using a different *Sf3b1^K700E/+^* donor mouse.

**Figure 3:**
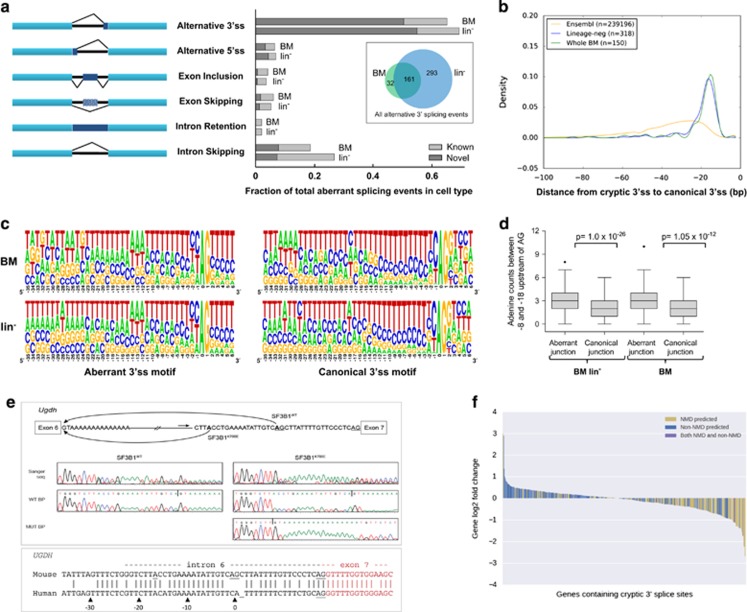
Aberrant 3′ splicing is the dominant abnormality associated with Sf3b1-K700E in bone marrow cells. (**a**) Types and relative frequencies of aberrant splicing events in *Sf3b1*^*K700E/+*^ vs *WT* BM and lin^*−*^ cells. Most are alternative 3′ splicing events, of which many are shared between BM and lin^−^ cells (Venn diagram). (**b**) Distribution of distances of aberrant 3′ss (AG) from the downstream canonical 3′ss in *Sf3b1*^*K700E/+*^ BM and lin^*−*^ RNA. This distance is significantly shorter than that of known cryptic ss annotated in Ensembl (all mouse junctions for the first AAG, TAG or CAG within 100 nt upstream from the canonical 3' splice site are considered). (**c**) Motif frequency plots for *Sf3b1*^*K700E/+*^ BM and lin^*−*^ samples contrasting aberrant and canonical 3′ ss sequences, showing an enrichment of adenines in the former. (**d**) Significant enrichment in adenines in positions -8 to -18 upstream of aberrant 3′ ss (AG) compared to the canonical junctions from *Sf3b1*^*K700E/+*^ BM and lin^*−*^ cells. (**e**) Lariat sequencing of the aberrant splice site of the *Ugdh* gene in *Sf3b1*^*K700E/+*^ mice, highlighting the location of the aberrant branch point (upper panel; the top trace is the raw data from Sanger sequencing and bottom is deconvoluted data from Mutation Surveyor). The gene is not misspliced at this locus in human SF3B1-mutant cancers as it does not retain the AG dinucleotide required for aberrant splicing (lower panel). (**f**) Association between aberrant splicing and gene expression. Transcripts that are NMD-predicted are significantly downregulated compared to transcripts that are non-NMD predicted (p = 3.92  ×  10^*−*13^, Kruskal *H* test).

Since *SF3B1* mutations affect pre-mRNA splicing and consequently gene expression,^13^ we performed mRNA sequencing from whole bone marrow (BM) and lin^*−*^ progenitors of *Sf3b1*^*K700E/+*^ and *WT* animals. Firstly, we observed that in *Sf3b1*^*K700E/+*^ mice transcript levels of the mutant (K700E) and wild-type *Sf3b1* mRNAs were very similar (Supplementary Table 3), confirming that expression from the targeted allele was not altered by genetic modification of the locus. We next sought to identify aberrant splicing events associated with *Sf3b1-K700E* using splice site analysis of RNAseq data from *Sf3b1*^*K700E/+*^ and *WT* BM cells as we described before^13^ (also see Materials and Methods). Compared to *WT* we identified 719 aberrant events in lin^*−*^ and 293 in whole BM cells from *Sf3b1*^*K700E/+*^ mice. The dominant type of aberrant splicing event was increased usage of cryptic 3′ splice sites (3′ ss) (Figure 3a), the majority of which were novel, that is, not reported in current transcriptome annotations. Aberrant splice sites were located within a narrow window of 15–24 nucleotides (nt) upstream from the canonical 3′ ss (Figure 3b) and associated with sequence features, including a shorter polypyrimidine tract and an enrichment of adenines at positions -8 to -18 upstream of the cryptic 3′ ss (Figures 3c and d), as described for human cancers with *SF3B1* mutations.^13^ Since SF3B1 is known to play a major role in U2 snRNP recruitment to the splicing branchpoint (BP), we performed lariat sequencing to identify BPs in aberrantly spliced mouse mRNAs. One example we identified was the *Ugdh* mRNA, for which the BP was located 16 nt upstream of the aberrant AG splice acceptor site that notably was not conserved in the human genome (Figure 3e). The *Get4* gene showed similar features (Supplementary Figure 6). These observations demonstrate that, as shown recently for human cancers,^11^ there is a distinct usage of cryptic BPs in cells expressing mutant SF3B1 in our mouse model.

Reconstruction of annotated transcripts that used aberrant 3′ splice sites in the lin^*−*^ RNAseq dataset revealed that approximately 42% of affected genes gain a premature termination codon in all isoforms, which is predicted to result in mRNA degradation through the nonsense-mediated decay (NMD) pathway. Genes for which NMD was predicted (*n* *=* 190) showed significantly lower mRNA expression levels than those for which NMD was not predicted (*n* *=* 225) (*P* = 3.92  ×  10^*−*13^, Kruskal *H* test, Figure 3f). Furthermore, looking at the transcriptome as a whole, we found that most (408 of 511) differentially expressed genes in lin^−^ cells were downregulated (discussed below). These findings demonstrate that *Sf3b1-K700E* is associated with abnormal splicing in hematopoietic stem/progenitor cells, primarily through the use of aberrant 3′-ss that are thought to result in NMD, and this is associated with significant downregulation of a large number of mRNAs.

Unbiased gene set enrichment analysis (GSEA) of the genes differentially expressed in *Sf3b1*^*K700E/+*^ revealed an enrichment of genes involved in RNA processing, splicing and transcription. Interestingly, similar enrichment was observed in human MDS^11^ (Supplementary Figure 7).

**Figure 4:**
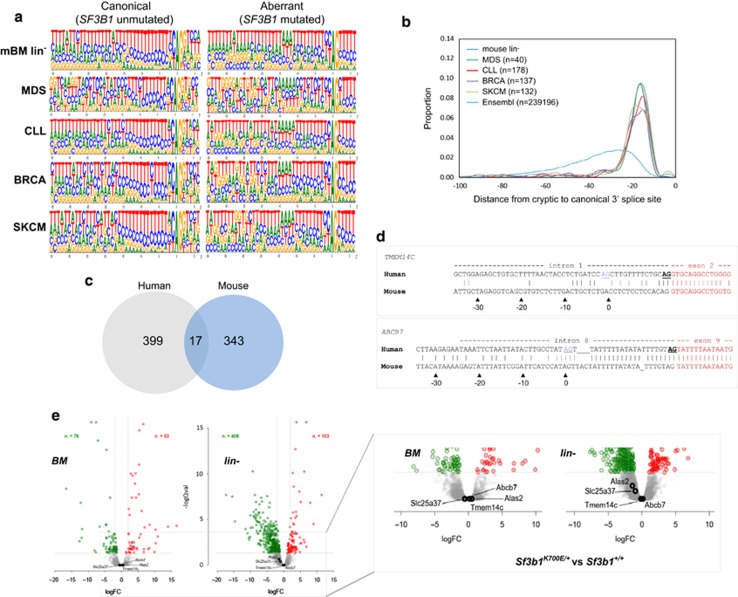
Mouse and human SF3B1-K700E share aberrant splicing properties, but affect different mRNA transcripts. (**a)** Comparison of consensus sequences upstream of aberrant and canonical 3′ ss shows a striking preservation of these motifs between mouse *Sf3b1*^*K700E/+*^ lin^*−*^ cells and several human *SF3B1*-mutant cancers. (**b**) Distribution of distances of aberrant 3′ss (AG) from the downstream canonical AG is also strikingly similar between mouse *Sf3b1*^*K700E/+*^ lin^*−*^ cells and human cancer samples. (**c**) By contrast, a comparison of genes found to be aberrantly spliced in human vs mouse samples shows minimal overlap. (**d**) Poor sequence preservation in the mouse of the aberrant splice sites affecting human genes *TMEM14C* and *ABCB7* that are thought to trigger the formation of ring sideroblasts in MDS. (**e**) Volcano plots comparing mRNA expression between *Sf3b1*^*K700E/+*^ vs *Sf3b1*^*+/+*^ BM and lin^*−*^ cells. The mRNA expression of genes *Slc25a37*, *Tmem14c*, *Alas2* and *Abcb7*, thought to be involved in the formation of ring sideroblasts, is not significantly altered by the K700E mutation in mice. The number of aberrant events used to generate images in **a** and **b** is indicated in **b** (brackets). BRCA, breast cancer; CLL, chronic lymphocytic leukemia; MDS, myelodysplastic syndromes; SKCM, skin cutaneous melanoma.

Broad transcriptomic changes were observed in human cancers with *SF3B1* mutations including MDS,^11, 27^ and we recently described the aberrant splicing patterns in such cancers.^13^ Here we perform a similar analysis on RNAseq data from *Sf3b1*^*K700E/+*^ mice and report that for aberrant 3′ splice sites with upstream cryptic AGs, splicing abnormalities are strikingly similar to those seen in human cancer samples, including the aberrant splice site consensus sequence (Figure 4a) and distance of aberrant 3′ splice sites from the canonical ones (Figure 4b). However, despite these striking similarities, there was minimal overlap between aberrantly spliced genes in mouse vs human cells. In fact, comparing the two datasets we found that only ~5% (17/360) of the aberrantly spliced genes in mouse *Sf3b1*^*K700E/+*^ lin^*−*^ cells were also aberrantly spliced in human samples (Figure 4c). To look at this more closely, we compared the nucleotide sequences at the location of the aberrant 3′ splice sites identified in human *SF3B1*-mutant cancers,^11, 13^ including MDS, and those identified in our *Sf3b1*^*K700E/+*^ mouse BM and lin^*−*^ cells with the equivalent positions in the genome of the other species, examining the conservation of the invariant AG dinucleotides at the splice sites and that of the upstream 35nt. We first examined genes thought to have a role in the formation of ring sideroblasts in human *SF3B1*-mutant MDS, namely *TMEM14C*^25^ and *ABCB7*,^28, 29^ for which aberrant 3′ splicing was observed in humans, but not in *Sf3b1*^*K700E/+*^ mice. The human *TMEM14C* aberrant splice site AG was not conserved in mice, while for *ABCB7*, although the AG dinucleotide was conserved, it was located 2 nt upstream and was preceded by a poorly conserved sequence that deviated significantly from the aberrant splicing consensus (Figure 4d). We then looked at the 360 canonical junctions with aberrant upstream 3′ splice sites in *Sf3b1*^*K700E/+*^ lin^*−*^ cells and found that 268 were conserved in the human genome. Of the 268 human junctions only 125 contained both a potential cryptic AG in the range preferred by *SF3B1/Sf3b1* mutants (-5 to -20nt) and a potential branchpoint adenosine at -8 to -18 from the cryptic AG, indicating that less than 35% of mouse junctions with aberrant 3′ splice sites had the hypothetical capacity for aberrant splicing in human cells. Of these 125 potential aberrant junctions, only 53 (14.7% of total) were found to be spliced in any human MDS sample, indicating that the limited conservation between mouse and human intronic sequences is responsible for the differences in misspliced mRNAs in the two species. We also noted that both *Tmem14c* and *Abcb7* as well as the *Alas2* (heme biosynthesis) and *Slc25a37* (mitochondrial iron importer) genes that are downregulated in human *SF3B1*-mutant MDS^11^ did not display altered expression in *Sf3b1*^*K700E/+*^ lin^*−*^ or whole BM cells (Figure 4e).

## Discussion

In order to understand the molecular and phenotypic consequences of *SF3B1* mutations on hematopoiesis and their role in the pathogenesis of clonal blood disorders, we generated a knock-in conditional mouse model of *SF3B1*-K700E, the most common mutation in human MDS. Our conditional allele preserved wild-type *Sf3b1* expression, thus avoiding any confounding effects of reduced *Sf3b1* levels on hematopoiesis^11, 30^ or other developmental effects. Conditional activation by *Mx1-Cre* of the *Sf3b1*-K700E mutation in *Sf3b1*^*flox−K700E/+*^ mice then enabled direct comparison to wild-type (*WT*) isogenic hematopoietic progenitors at the molecular and phenotypic level. At the molecular level, we report that the splicing defect seen in *Sf3b1*^*K700E/+*^ hematopoietic progenitors closely mimicked that described in human MDS, in sharing a strikingly similar splice-site consensus sequence and distance from the aberrant splice site AG. Furthermore, as was recently described for human cancer samples,^31, 32^ we show that aberrant BP recognition is central to the disrupted function of mutant SF3B1 in our mice. However, despite the closely similar properties of human and mouse SF3B1 mutants, we found that misspliced transcripts differed significantly between mouse and human as a result of the relatively poor interspecies conservation of intronic sequences able to function as aberrant splice sites. Interestingly, a recent study describing a murine model of *U2AF1*-S34F, another MDS-associated splicing gene mutation associated with aberrant 3′ splice acceptor site recognition, also showed limited mouse–human overlap of mispliced transcripts.^33^ The mouse–human overlap appeared greater for a mouse model of *SRSF2* mutations (*SRSF2*-P95H), another commonly mutated gene in human MDS, possibly because the splicing aberration involves alternative exonic sequences, which are more likely than intronic ones to be conserved.^34^

With regard to erythropoiesis, *Sf3b1*^*K700E/+*^ mice showed impaired terminal erythroid differentiation, associated with progressive normocytic anemia without ring sideroblasts. In human *SF3B1*-mutant MDS, ring sideroblasts are thought to arise as a result of aberrant splicing of key genes involved in heme biosynthesis, such as *ABCB7, TMEM14C, ALAS2* and *SLC25A37*.^11, 25, 30, 35^ In keeping with the mouse–human differences in target transcripts, we did not find the splicing or expression levels of any of these genes to be noticeably disrupted by *Sf3b1*-K700E, and, consistent with this, did not identify ring sideroblasts in the bone marrow of mutant mice. Of note, ring sideroblasts were not observed in single-gene knock-out mice for either *Alas2*^36^ or *Abcb7*,^37^ suggesting that sideroblast formation may require simultaneous reduction in the levels of multiple proteins involved in both heme biosynthesis (ALAS2, TMEM14C) and mitochondrial iron transport (ABCB7 and SLC25A37), and may therefore be difficult to replicate in single-gene targeting models. In this light, the development of anemia in *Sf3b1*^*K700E/+*^ mice may be at least in part due to globally disrupted splicing rather than effects on particular genes. Aberrant splicing leads to the introduction of premature termination codons, which can in turn lead to the generation of truncated proteins with potential toxic effects on cells, leading in this instance to anemia and impaired iron utilization manifested as excess hemosiderin deposition in the bone marrow of *Sf3b1*^*K700E/+*^ mice. An alternative or additional explanation may reside in the fact that both mouse and human cells displayed aberrant expression of genes involved in splicing and RNA processing itself (Supplementary Figure 7), which may be a response to, rather than a result of, globally disrupted splicing. In fact there is evidence that when their intracellular concentrations become too high, some splicing factors regulate their own expression by targeting their mRNAs for NMD.^38^

Beyond erythropoiesis, *Sf3b1*^*K700E/+*^ mice had reduced numbers of HSCs and displayed a myeloid cell bias. Furthermore, investigation of the self-renewal potential of *Sf3b1*^*K700E/+*^ HSCs as determined by their repopulating ability in competitive transplantation assays into either young or old recipient mice revealed a fitness disadvantage of mutant over wild-type HSCs. This contrasts with observations that mutant *SF3B1* can drive clonal hematopoiesis and can even be the sole identifiable driver mutation in human MDS.^7, 9^ However, observations that *SF3B1* mutations appear to selectively impart a clonal advantage on HSCs of elderly, but not young, individuals suggest that the phenomenon is context dependent even in humans.^26^ Furthermore, a lack of clonal HSC advantage was a feature of other mouse models of MDS-associated mutations, including *ASXL1*,^39^ *U2AF1*^33^ and *SRSF2*,^34^ all of which can also drive clonal hematopoiesis in humans,^40, 41^ proposing that factors other than somatic mutations may be operative in the development of MDS.^26^

The fact that disruption of a process as central to cellular function as RNA splicing can have detrimental effects on cells is unsurprising and further augments the conundrum of how particular spliceosome gene mutations can instead impart a survival/fitness benefit upon human HSCs, albeit in a context-dependent manner.^8, 26^ This remains the least-well-understood feature of spliceosome gene mutations, and our data propose that the different phenotypic effects of spliceosome gene mutations in MDS (e.g. anemia, ring sideroblasts, clonal expansion) may have distinct molecular causes. Future studies aimed at deciphering the mechanisms underlying these phenotypes should exploit both the similarities and the differences in the transcriptional consequences of these mutations between mice and humans, as a means of identifying their respective etiologies.
