# Supplementary material for: Hemopoietic-specific Sf3b1-K700E knock-in mice display the splicing defect seen in human MDS but develop anemia without ring sideroblasts
Source: Leukemia. 2016 Oct 21;31(3):720–7. doi: 10.1038/leu.2016.251 (PMC5336192; doi:10.1038/leu.2016.251)
Supplement: Supplementary Figures [file leu2016251x1.doc]

## Supplementary figure legends

### Supplementary figure 1. Retrieval of the target regions and generation of a gateway-adapted intermediate plasmid

**a.** Genomic location of the *Sf3b1* target locus against assembly details and bacterial artificial chromosome (BAC) end pairs mapping to the area. Location of recombineering primers (G5, D3, U5, G3) is shown relative the Sf3b1 locus. A pool of BACs RP24-257N19 and RP24-121E21 was used to retrieve the genomic DNA of the murine Sf3b1 locus. **b**. Schematic representation of how the “U” and “G” cassettes, encoding resistance markers and gateway consensus sequences, were generated by PCR from template plasmids (gifts from W. Skarnes, Wellcome Trust Sanger Institute). **c.** Sequential steps used to generate the intermediate vector (not in scale) with relevant *Sf3b1* exons represented in grey. First, the recombineering plasmid is inserted by heat shock into competent cells harboring the BAC. Then, the “U” cassette is inserted into the BAC through a recombineering reaction. Subsequently, a second recombineering reaction is used to extract the relevant segment of the BAC into the gateway-adapted intermediate vector. Finally, a KpnI fragment encoding exons 15 and 16 of the Sf3b1 gene is removed by digestion and replaced by ligation with the same sequence discordant only for the A>G mutation encoding the p.K700E mutation (grey box with red text). Antibiotics used for selection are indicated: C = chloramphenicol, T = tetracycline, Z = zeomycin, A = ampicillin

### Supplementary figure 2. Generation of the Sf3b1-K700E targeting vector

**a**. Steps taken to remove the 3’ LoxP site (top) and insert the synthetic cDNA splice-trap cassette (bottom) into the pL1L2_BactP vector^16^. b. Steps taken to assemble the final targeting vector using gateway recombination. The negative selection plasmid backbone (pL3L4-DTA) contains L3 and L4 sites flanking the bacterial ccdB and cat genes followed by a diphtheria toxin A-chain (DTA) expression cassette under a PGK promoter. The vector was linearized with the AsiSI restriction enzyme prior to electroporation. Bact = Beta Actin promoter, NEO = Neomycin, pA = PolyA, ori = origin of replication, PGK= mouse phosphoglycerate kinase 1 gene promoter

Supplementary figure 3. **Detailed structure of the *Sf3b1^K700E^* alleles**

**a.** To preserve wild type (WT) *Sf3b1* expression from the targeted allele, a floxed cassette encoding exons 12-25 of the *Sf3b1* cDNA followed by an SV40 polyadenylation signal (pA) was inserted by homologous recombination into intron 11. As we had found that a contiguous cDNA of exons 12-25 was toxic to bacteria, we separated exons 12-15, 16-19 and 20-25 using native *Sf3b1* introns 15-16 and 19-20 as shown. Also, exons 12-15 and 16-19 were optimized for mouse codon usage. This construct was followed by a neomycin resistance cassette (NEO) flanked by FRT sites. Downstream of the neomycin cassette, at the native exon 15 (yellow), the K700 codon was modified (AAA>GAA) by site-directed mutagenesis introducing the K700E mutation. The gel image shows validation of correct ESC targeting by long-range PCR using primers located outside the targeting construct. **b.** The ***native*** *Sf3b1* locus with numbered exons and predicted RNA splicing pattern is shown at the top. The ***targeted*** allele is predicted to express a part codon-optimized *Sf3b1* mRNA coding for the wild type protein. The neomycin cassette was then excised by breeding with *Rosa26-Flpe* mice to generate the ***post-Flpe*** conditional allele (=***Sf3b1^flox-K700E^***). The ***Sf3b1^flox-K700E^*** mutation could then be activated by Cre-*loxP* recombination (***Post-Cre***). c/o = codon-optimized for mouse

### Supplementary figure 4. Molecular validation of the Sf3b1 allele

**a.** Schematic representation of the native (blue) and chimeric (blue and ocra) WT *Sf3b1* mRNAs with location of correspondingly color-coded primers used for validation. **b.** Results of RT-PCRs showing amplification of the chimeric (part codon-optimized) mRNA species specifically from correctly targeted ESC clones, demonstrating successful splicing around intronic regions in the splice-trap cassette. By contrast, the native mRNA is expressed in WT andf NIH3T3 cells. **c.** Schematic representation of the native *Sf3b1* locus and of the targeted locus after Flpe followed by Cre-mediated recombination events and location of primers used. The gel image shows that double recombined targeted ESCs give both the native (shorter) and the recombined (longer) bands. **d.** Capillary sequencing of cDNA from the same D9 clone, before (left) and after Cre-*loxP* recombination (right), highlighting the A>G mutation, encoding the K700E mutant codon.

**Supplementary figure 5. Additional phenotypic characterization of *Sf3b1*^K700E+/-^ mice**

**a.** Kaplan-Mayer plots showing no difference in the survival rate of *Sf3b1*^K700E+/-^ mice compared to WT. **b.** FACS analysis of bone marrow cells from *Sf3b1^K700E/+^* mice showed increased numbers of Gr1+/Mac1+ cells compared to WT controls.

**Supplementary figure 6**. **Identification of cryptic splicing branchpoints by lariat sequencing**

**a.** Location of canonical and cryptic/aberrant branchpoints (BP) and splice sites identified by lariat sequencing at the indicated gene intron **b.** Mapping of the different BPs using nested PCR analysis and sequencing of *Get4* intron 5 lariat in whole BM cells from *Sf3b1^+/+^* and *Sf3b1^K700E+/-^*samples. Canonical branchpoint (BP) "A" in bold/underlined, cryptic BP "A" or "G" in orange, canonical AG in blue and cryptic AG in red.

**Supplementary figure 7. Gene set enrichment analysis (GSEA) score curves.**

GSEA plots show enrichment for pathways involved in RNA splicing and processing from both mouse *Sf3b1^K700^*^/+^ BM and lin^-^ samples, as well as human MDS. Enrichment scores (ES) and q-values are indicated.
