# Supplementary figures and images for: Hemopoietic-specific Sf3b1-K700E knock-in mice display the splicing defect seen in human MDS but develop anemia without ring sideroblasts
Source: Leukemia. 2016 Oct 21;31(3):720–7. doi: 10.1038/leu.2016.251 (PMC5336192; doi:10.1038/leu.2016.251)

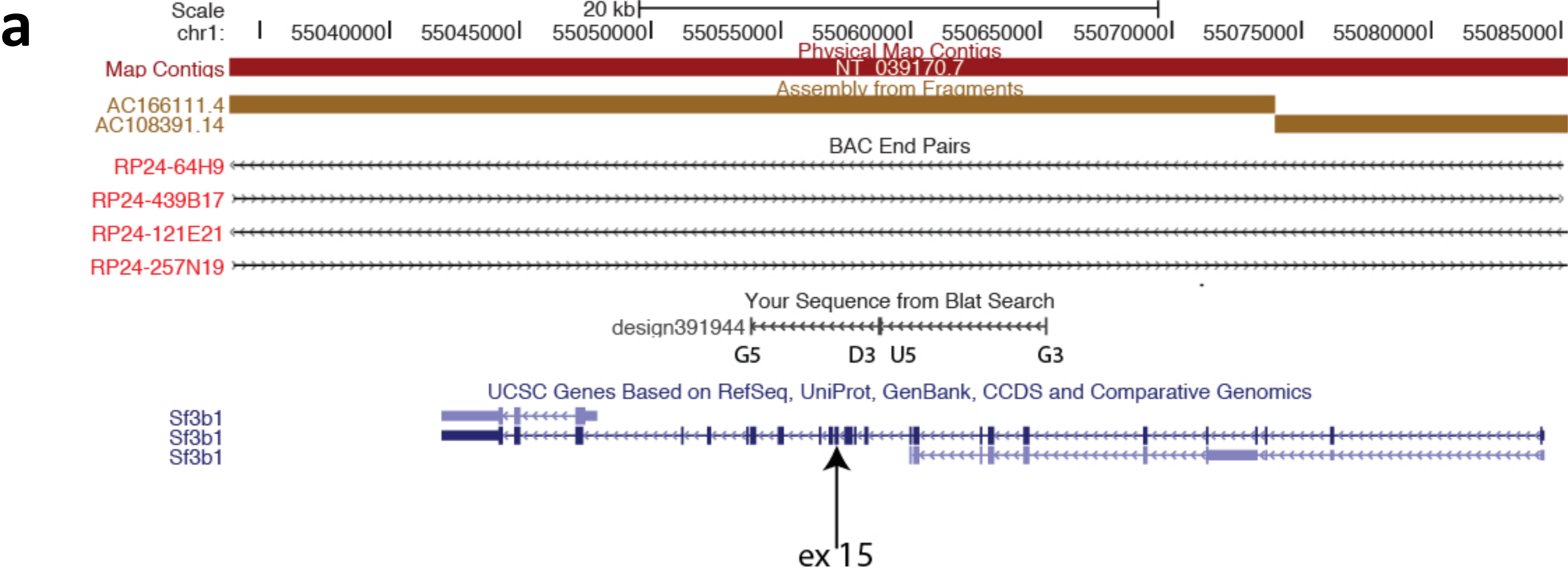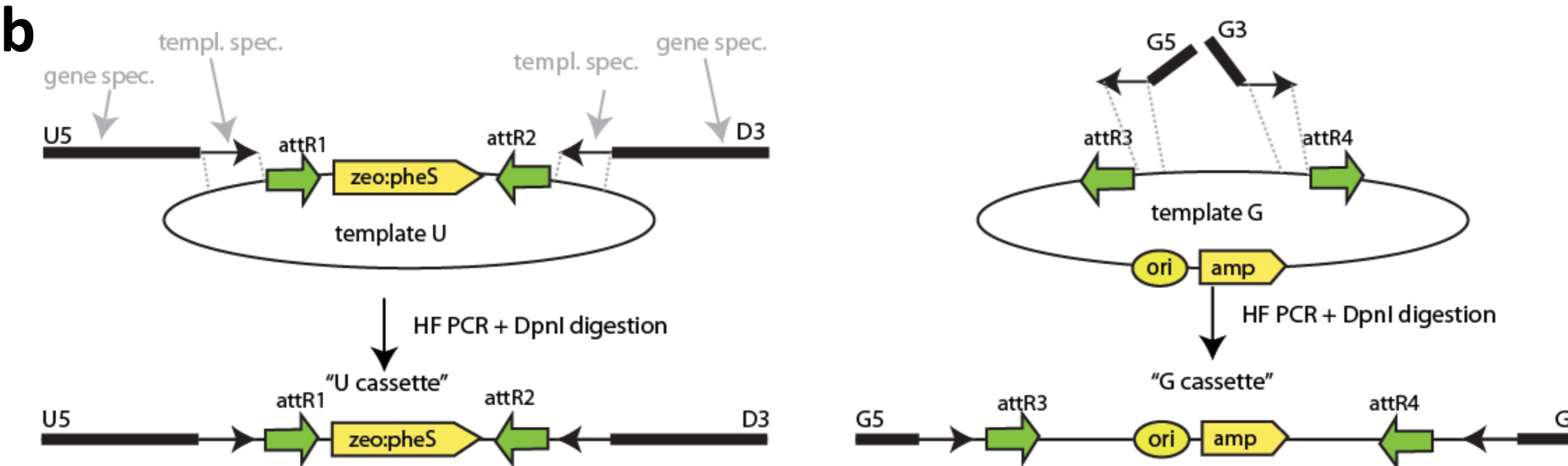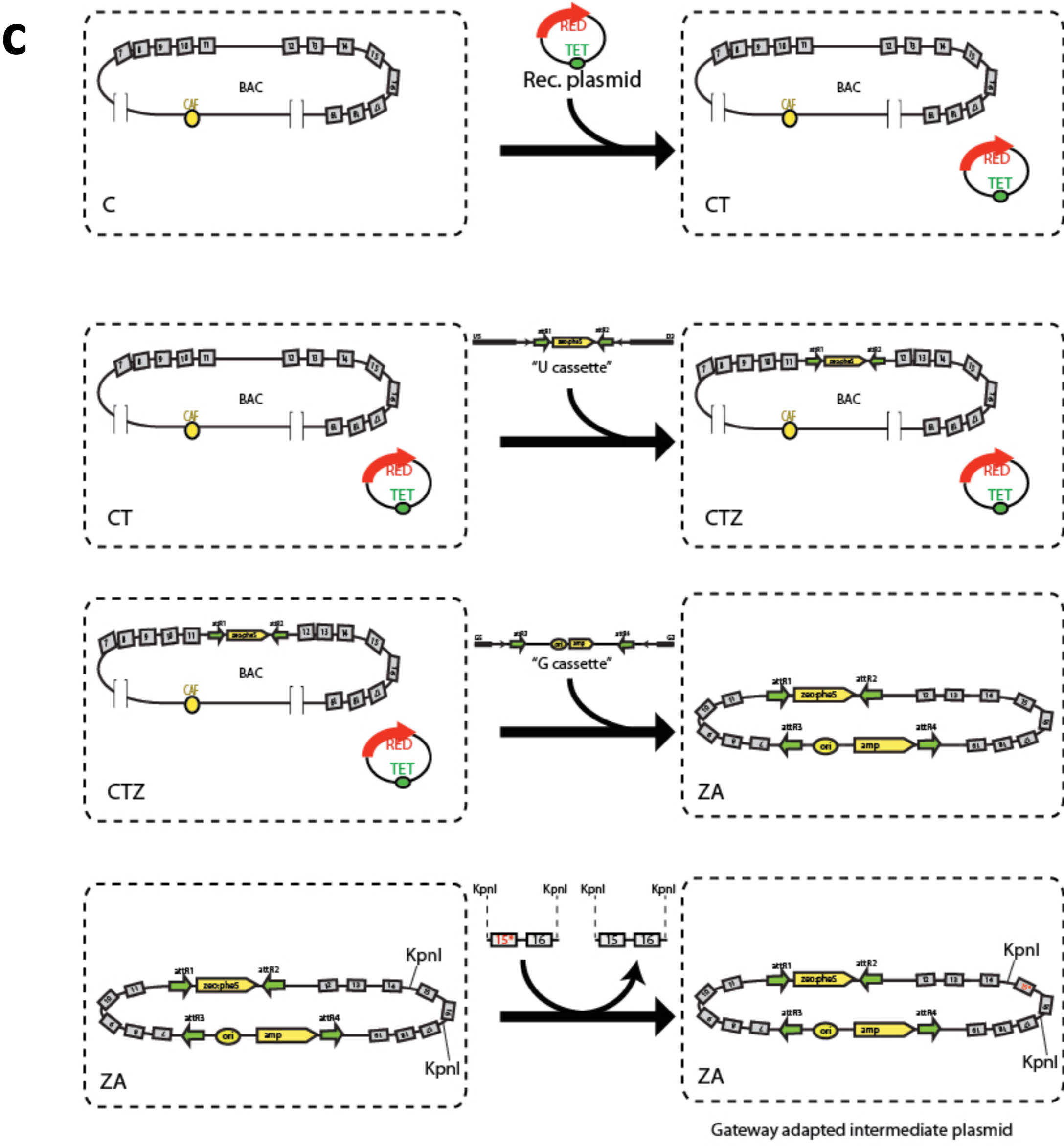

Supplementary figure 1

Supplement: Supplementary Figure 1 [file leu2016251x2.pdf]

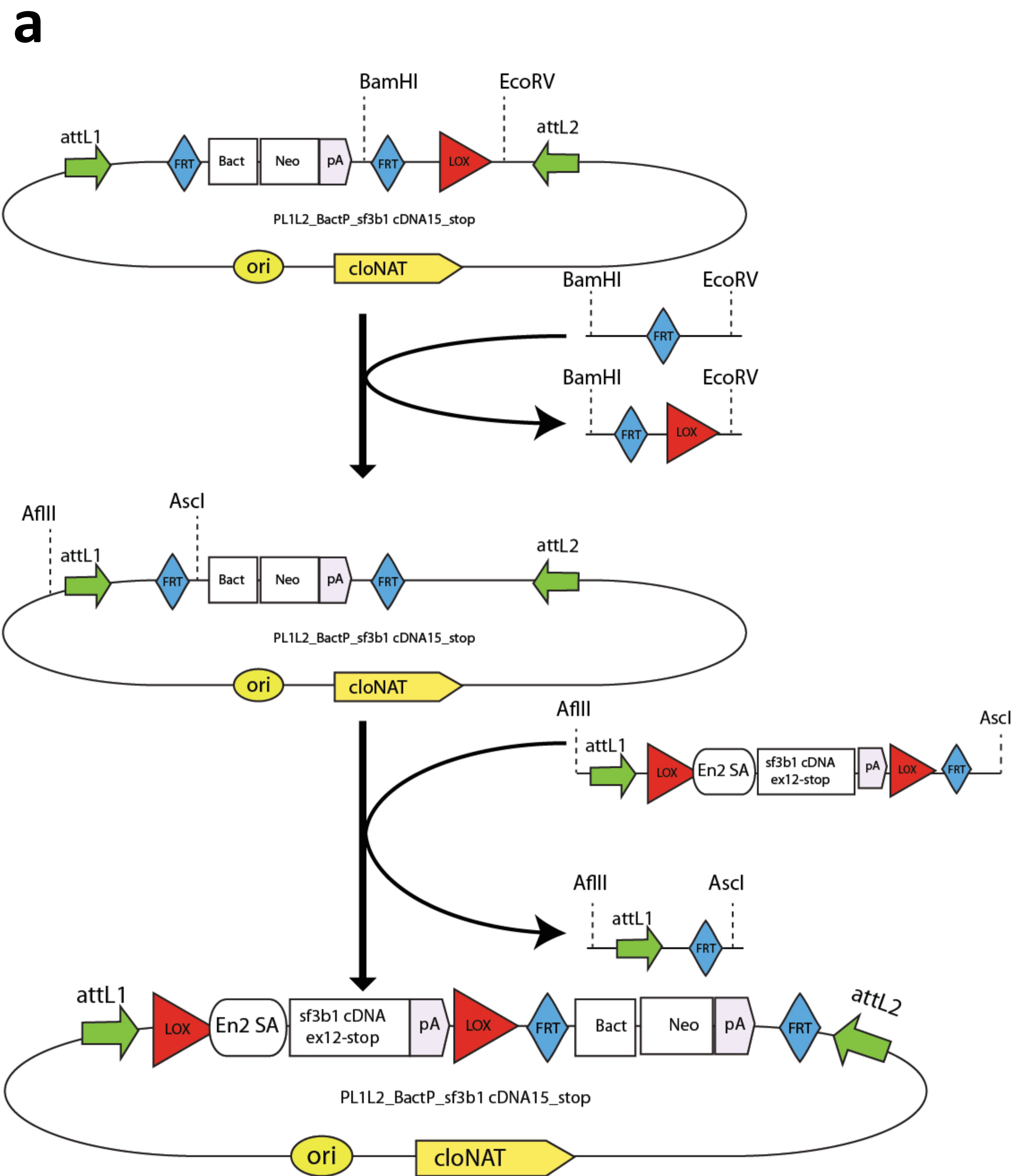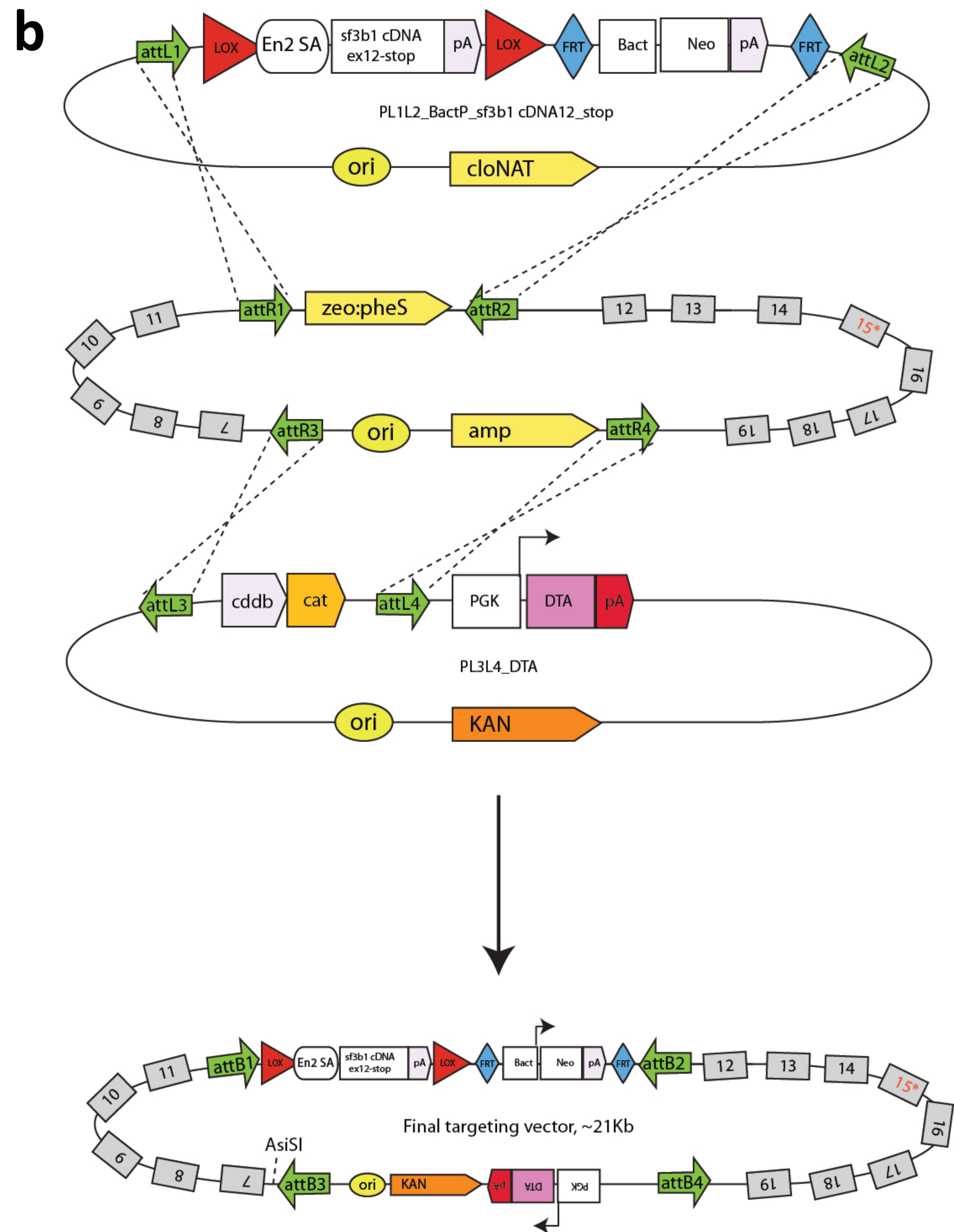

**Supplementary Figure 2**

Supplement: Supplementary Figure 2 [file leu2016251x3.pdf]

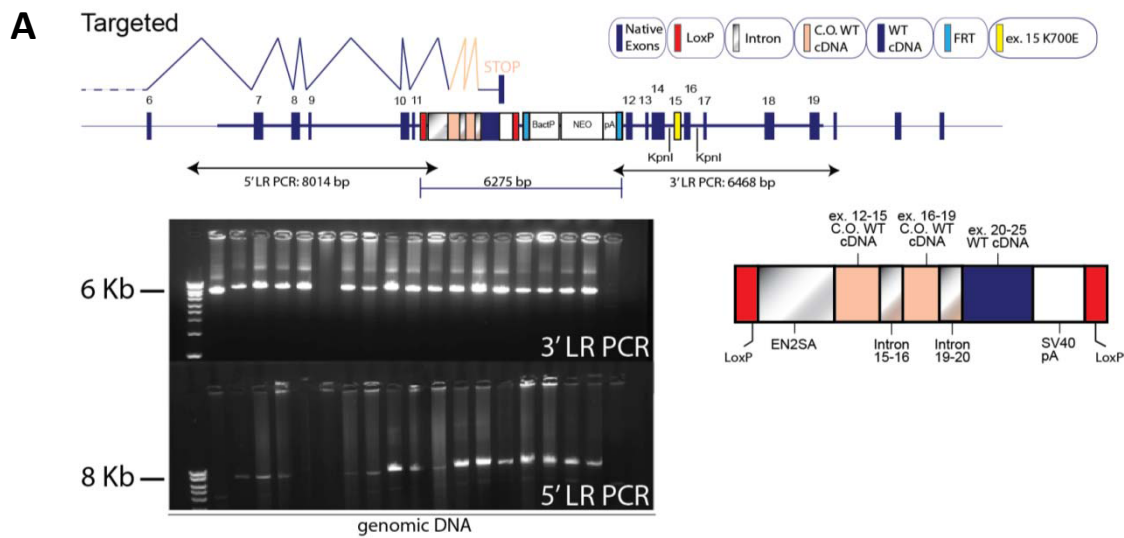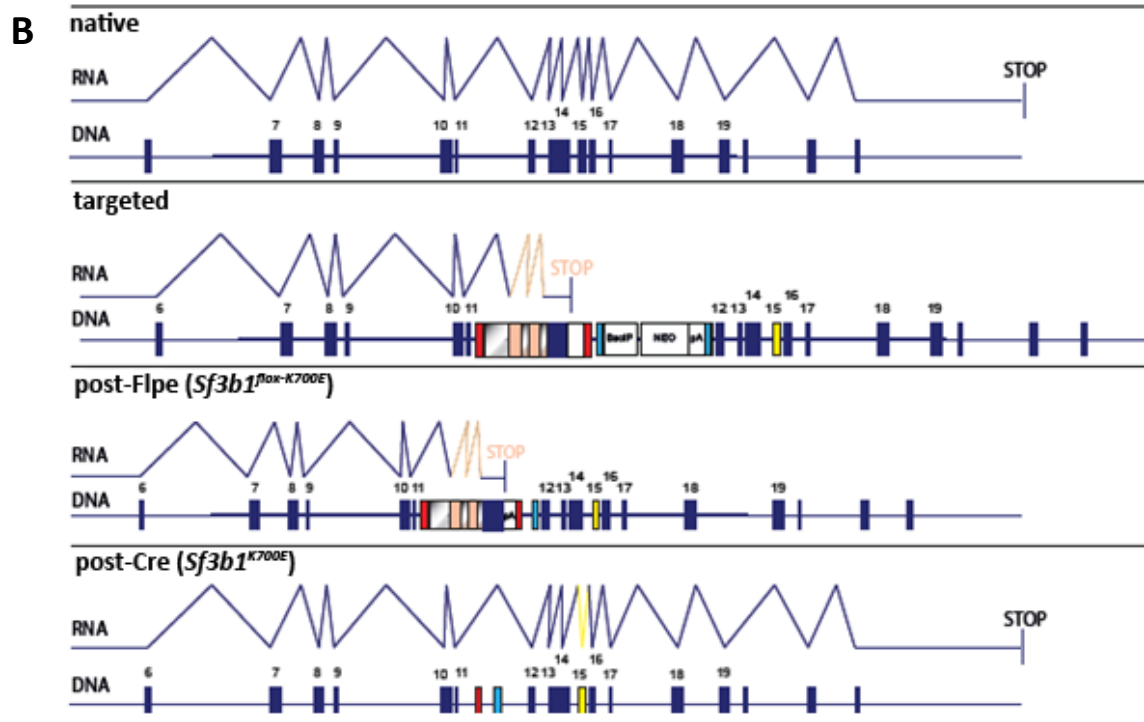

**Supplementary Figure 3**

Supplement: Supplementary Figure 3 [file leu2016251x4.pdf]

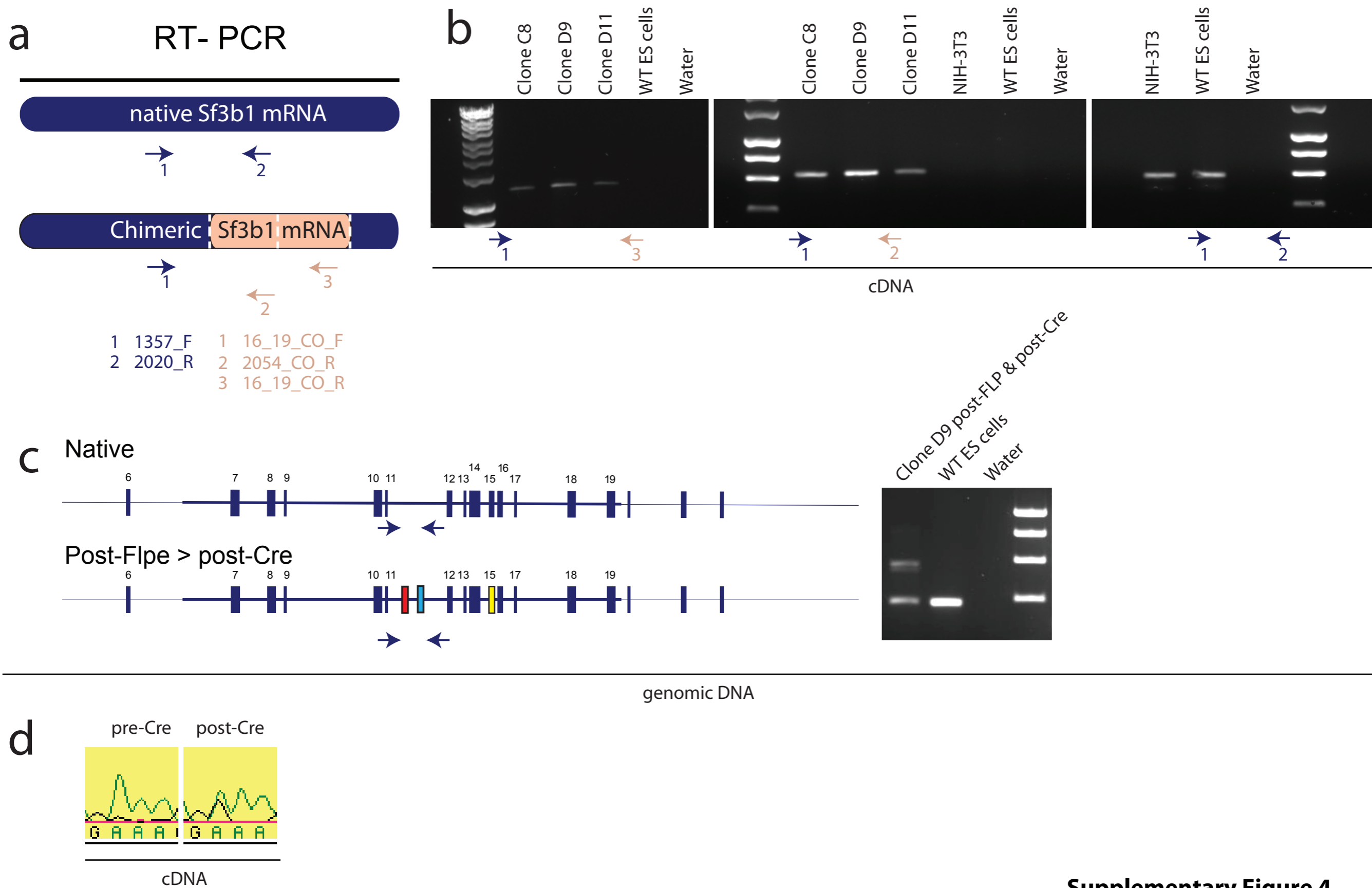

**Supplementary Figure 4**

Supplement: Supplementary Figure 4 [file leu2016251x5.pdf]

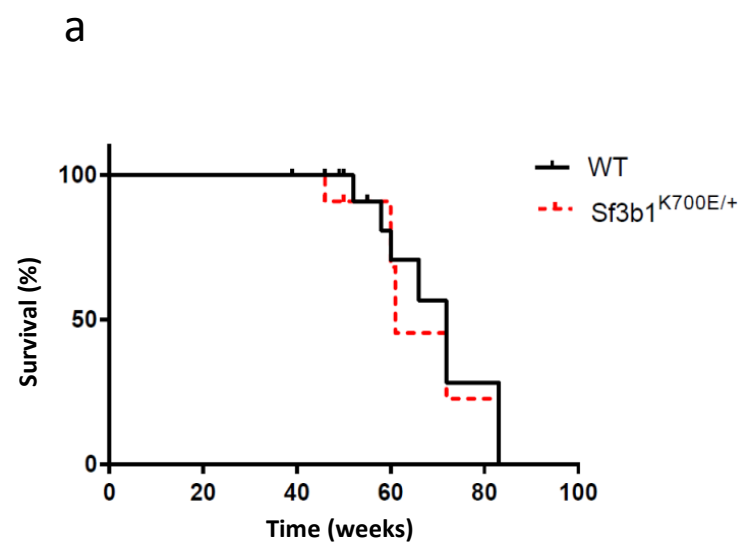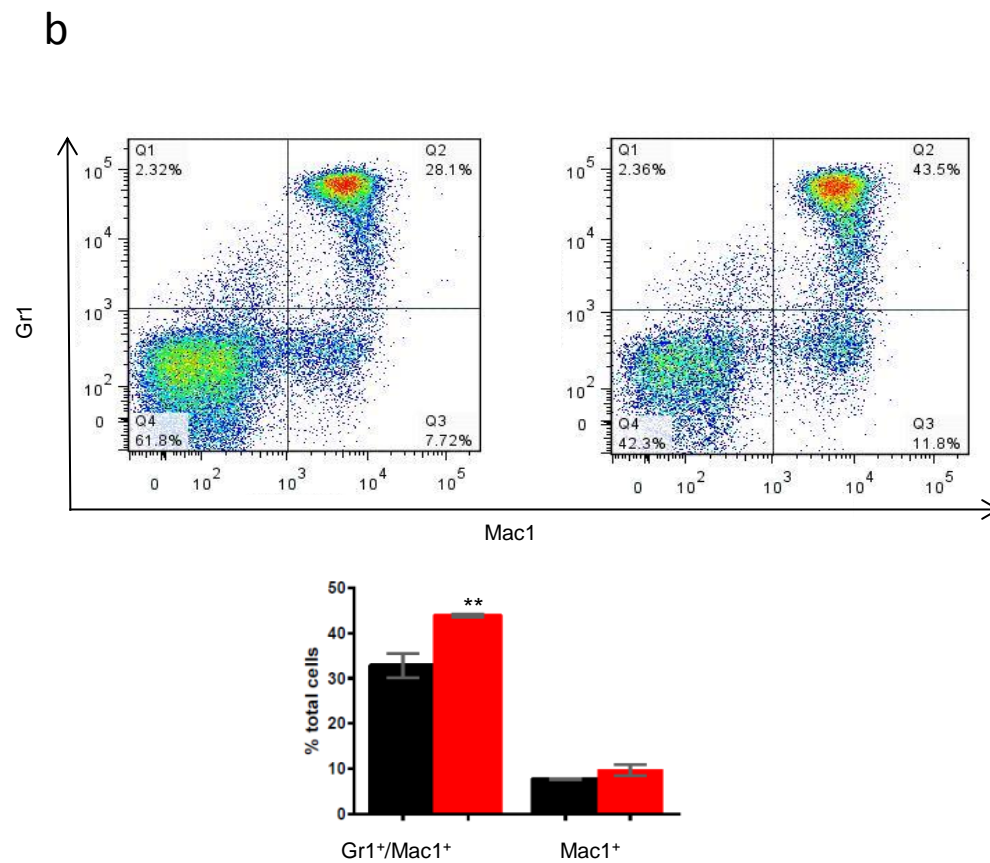

Supplementary Figure 5

Supplement: Supplementary Figure 5 [file leu2016251x6.pdf]
