# Supplementary material for: Hemopoietic-specific Sf3b1-K700E knock-in mice display the splicing defect seen in human MDS but develop anemia without ring sideroblasts
Source: Leukemia. 2016 Oct 21;31(3):720–7. doi: 10.1038/leu.2016.251 (PMC5336192; doi:10.1038/leu.2016.251)
Supplement: Supplementary Figure 6 [file leu2016251x7.pdf]

a

Gene	IVS	3' splice site
Ugdh	intron 6	CTTACCTGAAAATATTGTCAGCTTATTTTGTTCCCTCAG
Get4	intron 4	GCTCACGTGTGCCCATCTGCAGATTCTTTCCCTTCACAG

b

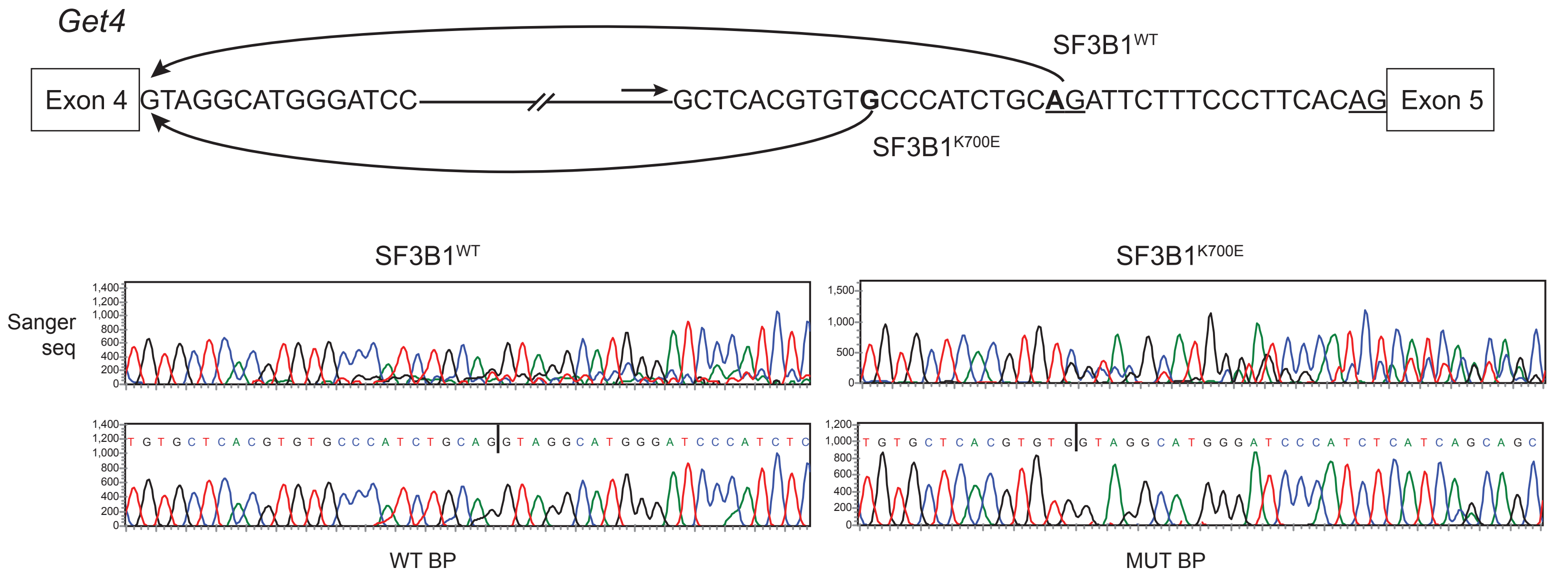

Supplementary figure 6
