# Supplementary material for: Hemopoietic-specific Sf3b1-K700E knock-in mice display the splicing defect seen in human MDS but develop anemia without ring sideroblasts
Source: Leukemia. 2016 Oct 21;31(3):720–7. doi: 10.1038/leu.2016.251 (PMC5336192; doi:10.1038/leu.2016.251)
Supplement: Supplementary Figure 7 [file leu2016251x8.pdf]

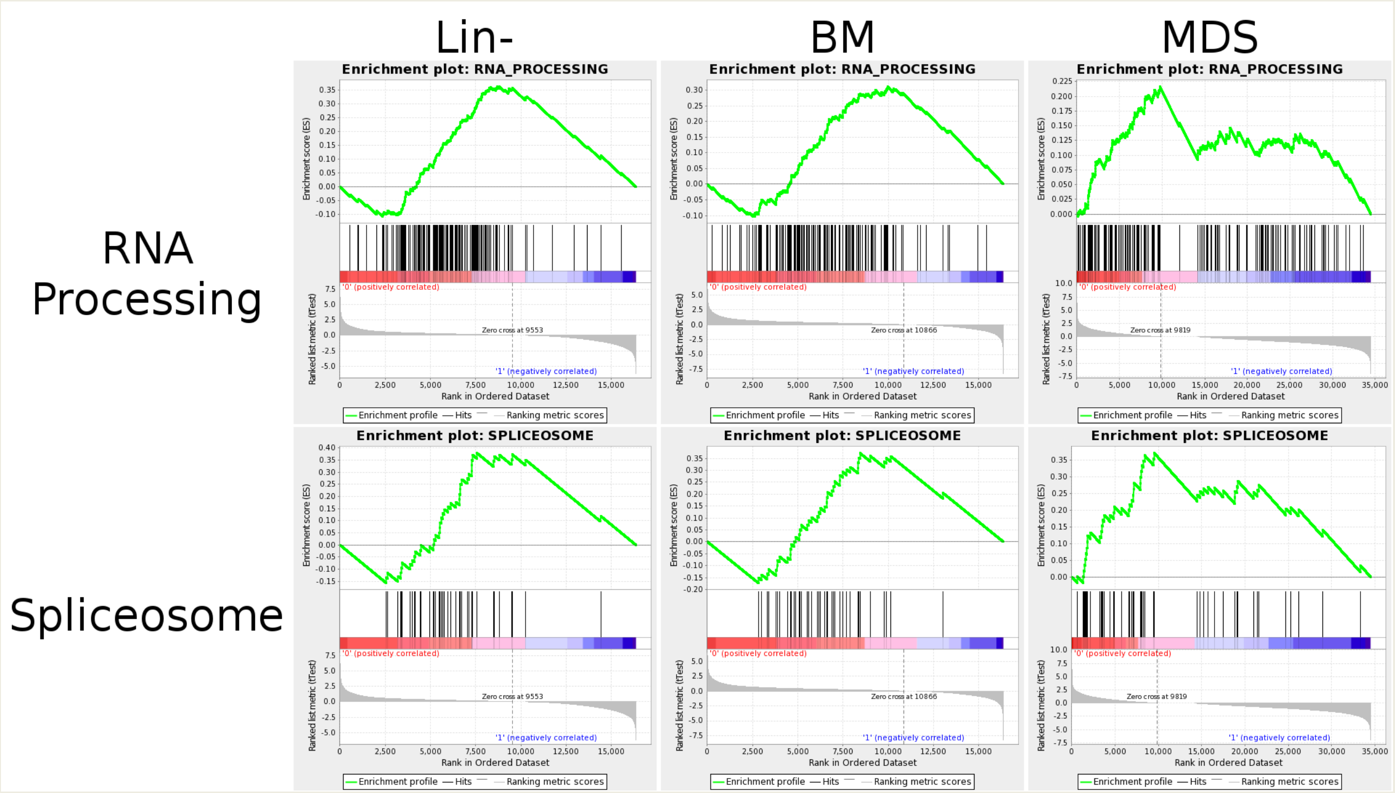

		LN	BM	MDS
Spliceosome	ES	0.37901253	0.37184516	0.37165445
	q-value	5.38E-005	5.46E-005	0
RNA Processing	ES	0.3625852	0.31063414	0.21633695
	q-value	0	0	0

Supplementary figure 7
