# Supplementary material for: Hemopoietic-specific Sf3b1-K700E knock-in mice display the splicing defect seen in human MDS but develop anemia without ring sideroblasts
Source: Leukemia. 2016 Oct 21;31(3):720–7. doi: 10.1038/leu.2016.251 (PMC5336192; doi:10.1038/leu.2016.251)
Supplement: Supplementary Table 1 [file leu2016251x9.pdf]

Gateway-adapted intermediate plasmid primers

gene specific + D3	ATGTTTATCCTGGAGCATGACCTACCTGTCCACTTGCACACATATCCTTG CCGCCTACTGCGACTATAGA
gene specific + U5	TGCCAGTACCTTTTACCCACTGAGGAAGGAGTCTTGCTAGCCCTCTTAAGAAGGCGCATAACGATACCAC
gene specific + G5	CCAGCAACCACATGGTGGCTCACAACCAACTGAAATGGGAGCTGATGCCCTCCTGTGTGAAATTGTTATCCGC
gene specific + G3	AAGGCTGAGAGTTCATAGGACCTGGCTGTTGGTAATCACTGTTCTTCCCACCACTGGCCGTCGTTTTACA

5′ and 3′ long range PCR primers

5LRPCR_F	CTCCACGCTTGCTCAGTGACGTGTGTACTC
5LRPCR_R	CAAGTGTGGAGGGTGGTGT
3LRPCR_F	TAATCAGCCATATCACATCTGTAGAGG
3LRPCR_R	GTCAGCAATGCGTCCAACAAGATC

ES clone validation PCR primers

1357_F	CCTTTGGGTGGTATGACTGG
2020_R	GGCTACGACAGCAAAAGCTC
16_19_co_F	TGGTTGATACCACCGTTGAA
2054_co_R	GAAACGGCAGCAGGCTC
16_19_co_R	TCAACGGTGGTATCAACCAG

ES clones validation post-exposure to FLP and Cre recombinases PCR primers

mmSf3b1_gDNA_25363F	AACAGAGATTCTTCCACCTTTCT
BactNEO_gen_R	GTGGCAGGTGAGGGCTTAGAGGTA

cDNA sequencing primers

mmsf3b1_1742f	CTTGAGGATCAAGAGCGACA
mm_Sf3b1_2400_R	TTGGCATATTCTGCATCCAT

Supplementary Table 1

Sequnces of primers used for generation of the Sf3b1 K700E targeting construct
