# Supplementary material for: Hemopoietic-specific Sf3b1-K700E knock-in mice display the splicing defect seen in human MDS but develop anemia without ring sideroblasts
Source: Leukemia. 2016 Oct 21;31(3):720–7. doi: 10.1038/leu.2016.251 (PMC5336192; doi:10.1038/leu.2016.251)
Supplement: Supplementary Table 2 [file leu2016251x10.pdf]

Ugdh
A (R): CAAACAAACACCCTCTTCCATAAA
B (F): TCACCATGTAGCTGCAAGAAA
C (R): TCACGGAGATTCACCAGTTTC
D (F): GGGAAGCAGTTAGTATGCACTTA

Get4
A (R): GAGGTCTATGACCAAAGCAGAG
B (F): AGGTGTGTCCAAGGCAAAT
C (R): AGGACAGAGACTGGAAAGGA
D (F): GCACACAAGCGTTAGGTAGA

SupplementaryTable 2
Primers used in lariat PCR for identification of aberrant RNA splicing branchpoints in selected mouse genes
