# Supplementary material for: Hemopoietic-specific Sf3b1-K700E knock-in mice display the splicing defect seen in human MDS but develop anemia without ring sideroblasts
Source: Leukemia. 2016 Oct 21;31(3):720–7. doi: 10.1038/leu.2016.251 (PMC5336192; doi:10.1038/leu.2016.251)
Supplement: Supplementary Table 3 [file leu2016251x11.pdf]

Sample ID	Aminoacid Change	Base Change	Allele Frequency	Coverage
BM1	p.K700E	chr1:55000367A>G	50.16%	1278
BM2	p.K700E	chr1:55000367A>G	50.90%	1167
BM3	p.K700E	chr1:55000367A>G	50.99%	1314
lin^- 1	p.K700E	chr1:55000367A>G	50.16%	1585
lin^- 2	p.K700E	chr1:55000367A>G	49.67%	1494
lin^- 3	p.K700E	chr1:55000367A>G	49.76%	1634

Supplementary Table 3

Mutant allele frequency in RNAseq data from whole bone marrow (BM) and lineage negative (lin^-) cells from three $Sf3b1^{K700E/+}$ mice
